# A method to analyze gene expression profiles from hippocampal neurons electrophysiologically recorded *in vivo*

**DOI:** 10.3389/fnins.2024.1360432

**Published:** 2024-04-17

**Authors:** Haruya Yagishita, Yasuhiro Go, Kazuki Okamoto, Nariko Arimura, Yuji Ikegaya, Takuya Sasaki

**Affiliations:** ^1^Department of Pharmacology, Graduate School of Pharmaceutical Sciences, Tohoku University, Sendai, Miyagi, Japan; ^2^Laboratory of Chemical Pharmacology, Graduate School of Pharmaceutical Sciences, The University of Tokyo, Tokyo, Japan; ^3^Graduate School of Information Science, University of Hyogo, Hyogo, Japan; ^4^Department of System Neuroscience, Division of Behavioral Development, National Institute for Physiological Sciences, National Institutes of Natural Sciences, Okazaki, Aichi, Japan; ^5^Cognitive Genomics Research Group, Exploratory Research Center on Life and Living Systems (ExCELLS), National Institutes of Natural Sciences, Okazaki, Aichi, Japan; ^6^Department of Neuroanatomy, Graduate School of Medicine, Juntendo University, Tokyo, Japan; ^7^Department of Cell Biology and Neuroscience, Graduate School of Medicine, Juntendo University, Bunkyo, Tokyo, Japan; ^8^Center for Information and Neural Networks, National Institute of Information and Communications Technology, Osaka, Japan; ^9^Institute for AI and Beyond, The University of Tokyo, Tokyo, Japan

**Keywords:** juxtacellular recording, single cell RNA sequencing, spike rise time, firing rate, burstiness

## Abstract

Hippocampal pyramidal neurons exhibit diverse spike patterns and gene expression profiles. However, their relationships with single neurons are not fully understood. In this study, we designed an electrophysiology-based experimental procedure to identify gene expression profiles using RNA sequencing of single hippocampal pyramidal neurons whose spike patterns were recorded in living mice. This technique involves a sequence of experiments consisting of *in vivo* juxtacellular recording and labeling, brain slicing, cell collection, and transcriptome analysis. We demonstrated that the expression levels of a subset of genes in individual hippocampal pyramidal neurons were significantly correlated with their spike burstiness, submillisecond-level spike rise times or spike rates, directly measured by *in vivo* electrophysiological recordings. Because this methodological approach can be applied across a wide range of brain regions, it is expected to contribute to studies on various neuronal heterogeneities to understand how physiological spike patterns are associated with gene expression profiles.

## Introduction

The hippocampus is composed of millions of excitatory pyramidal neurons, and their cooperative spike activity underlies information processing in episodic learning and memory (Scoville and Milner, 1957; Zola-Morgan et al., 1986). A number of physiological studies with large-scale multiunit recordings from the hippocampus have demonstrated that spike patterns (e.g., frequency, burstiness, inter-spike interval, synchronicity with other neurons) are not homogenous but are rather considerably variable across individual pyramidal neurons during spatial encoding and memory processing in living animals (Ylinen et al., 1995; Csicsvari et al., 2000; Mizuseki and Buzsaki, 2013), highlighting the need for further investigation of the physiological diversity of hippocampal pyramidal neurons.

However, evidence has accumulated that hippocampal pyramidal cells are highly heterogeneous and can be classified into distinct subpopulations based on their developmental processes (Cavalieri et al., 2021; Huszar et al., 2022), morphological characteristics (Graves et al., 2012; Thome et al., 2014), and gene expression profiles (Cembrowski et al., 2016; Habib et al., 2016; Saunders et al., 2018; Cembrowski and Spruston, 2019). Furthermore, recent advancements in spatial transcriptome analysis have enabled the evaluation of how the gene expression patterns of hippocampal neurons are spatially distributed in the hippocampal tissue (Rodriques et al., 2019; Stickels et al., 2021). A key question is how these heterogeneous molecular characteristics of hippocampal neurons are associated with their diverse physiological spike patterns.

Several techniques have been applied to neocortical neurons to address the issue of linking physiological and molecular characteristics. For example, *in vivo* two-photon calcium imaging to record activity patterns of neurons is combined with subsequent multiplexed fluorescent *in situ* hybridization (Xu et al., 2020; Bugeon et al., 2022) or sampling of the imaged neurons for single-cell RNA sequencing (Lee et al., 2019; Liu et al., 2020; O'Toole et al., 2023). Although these imaging-based approaches are effective for the analysis of neocortical neurons, they are not well-suited for analyzing hippocampal neurons, where the excitation light for optical imaging is less accessible, and the synchronization of spike patterns with extracellular oscillations on a millisecond level plays a crucial role in learning and memory.

To target the hippocampal neurons, we used *in vivo* juxtacellular recording (Pinault, 1994, 1996; Oyama et al., 2013; Dempsey et al., 2015), an electrophysiology-based single-cell spike recording technique, to reveal the link between spike patterns and gene expression patterns. *In vivo* juxtacellular recording is a unique method to locate the recorded neurons and is therefore useful in combination with other methods such as morphological analysis (Hodapp et al., 2022) and *in situ* hybridization (Mallet et al., 2006). Our procedures involved juxtacellular recording from hippocampal neurons, followed by a series of experiments, including cell labeling, cell sampling (Hempel et al., 2007), and single-cell RNA sequencing (Sasagawa et al., 2018). Here, we demonstrate that several genes are indeed correlated with the spike patterns of hippocampal neurons recorded from a head-fixed mouse.

## Materials and methods

### Experimental animals

All experiments were performed with the approval of the Experimental Animal Ethics Committee at the University of Tokyo (approval number: P29-7) and the Committee on Animal Experiments at Tohoku University (approval number: 2022 PhA-004) and according to the NIH guidelines for the care and use of animals. A total of 25 male ICR mice (21 days old; SLC, Shizuoka, Japan) were used. The mice were housed on a 12-h light/12-h dark schedule with lights off at 8:00 PM. Food and water were readily available.

### Surgery

All the mice were anesthetized with urethane (2.25 g/kg, i.p.) (Nishimura et al., 2020, 2021). In a previous study, hippocampal neurons were active under urethane-induced anesthesia (Yagishita et al., 2020). After confirming that there was no righting reflex in response to hind limb pinching, the mice were fixed in a stereotaxic instrument (Narishige, Tokyo, Japan) with two ear bars and a nose clamp. An incision was made along the midline of the scalp, from the area between the eyes to the back of the head, and the periosteal soft tissue within the incised area was removed. An area for the cranial window (1.5 × 2.0 mm^2^; 1.8 mm posterior to the bregma and 1.8 mm ventrolateral to the sagittal suture) was marked. The skull surface outside the cranial window was coated with dental resin, and a plastic plate (designed and printed using a 3D printer) was fixed to the head using dental cement. The mice were moved to another stereotaxic instrument (O’Hara & Co., Ltd., Tokyo, Japan), a craniotomy was performed to create a rectangular hole, and the dura was removed. The cranial window was covered with phosphate-buffered saline (PBS; pH 7.4) until glass pipettes were inserted.

### *In vivo* juxtacellular recordings

Through the window, a borosilicate glass pipette (4.5–16.1 MΩ) was inserted at 100 μm/s to a depth of 900 μm from the brain surface, and the PBS on the cranial window was replaced with 1.7% agar. The electrode was slowly lowered at 0.2 μm/s into the hippocampus, and a juxtacellular recording was obtained from a neuron in the CA1 stratum pyramidale. The intra-electrode solution consisted of the following reagents: a fluorescent dye (1 mM Alexa 488 hydrazide and/or 1 mM Alexa 594 hydrazide), 1.5% biocytin, 135 mM NaCl, 5.4 mM KCl, 5 mM HEPES, 1.8 mM CaCl_2_, and 1 mM MgCl_2_. The solution was adjusted to pH 7.2–7.3 and 285–300 mOsm and filter-sterilized through a 0.2-μm filter. Extracellularly recorded signals were amplified with an ELS-03XS amplifier (NPI Electronic, Tamm, Germany), digitized at 20 kHz with Axon Digidata 1550 B (Molecular Devices, San Jose, CA, USA), and analyzed using pCLAMP 12.1 software (Molecular Devices). Juxtacellular recordings were maintained under two criteria: the electrode resistance was <2.5 times the baseline that was observed at the beginning of recordings; the amplitude of spike waveforms was >1.5 mV. During the period that met these criteria, recordings were obtained for up to a maximum of 30 min. The cell labeling was then performed as described in the next paragraph. When the downward components of the spikes disappeared during recordings, recordings were immediately terminated, resulting in a recording duration of less than 30 min, and the cell labeling was then performed. No significant correlations were found between recording durations and expression levels of 25 metagenes (*n* = 40 cells, *p* > 0.05, Spearman’s correlation).

We attempted to insert an electrode into the brain with the two Alexa fluorescent dyes three times for each hemisphere, resulting in up to six attempts in total in one mouse. Based on our skills, the success rate of obtaining a juxtacellular recording was approximately 60%.

### Cell labeling

After recording, the recorded neurons were labeled with an Alexa fluorophore through electroporation using one of the following methods. A current-based method (Pinault, 1994, 1996) was performed by injecting rectangular current pulses (5–20 nA, 500 ms on/off or 250 ms on/off) for 3–10 min. The amplitude of the current for each neuron was adjusted such that the neuron emitted spikes in response to the current pulses. In addition to the evoked spikes, fluctuations in the baseline voltages were enhanced during current injection. A voltage-based method (Oyama et al., 2013; Dempsey et al., 2015) was performed by injecting 300 monophasic pulse trains (−10 V, 0.5 ms) at 100 Hz. Successful labeling was signaled by the transient broadening of evoked spikes and the disappearance of the downward components of the evoked spikes. Both methods successfully labeled a single neuron in each experiment. Current- and voltage-based methods were applied to 8 neurons in 7 mice and 32 neurons in 19 mice, respectively. No significant differences were detected for all of the 25 metagenes tested (*n* = 8 and 32 cells, *p* > 0.05, Student’s *t*-test).

### Acute slice preparation

After cell labeling, the mice were decapitated under anesthesia. The brains were removed quickly, and coronal hippocampal slices (200–300 μm thick) were prepared using a vibratome in ice-cold, oxygenated modified artificial cerebrospinal fluid (modified ACSF) (Sasaki et al., 2011), which consisted of 222.1 mM sucrose, 27 mM NaHCO_3_, 1.4 mM NaH_2_PO_4_, 2.5 mM KCl, 1 mM CaCl_2_, 7 mM MgSO_4_, and 0.5 mM ascorbic acid. The slices were incubated in oxygenated ACSF, which consisted of 127 mM NaCl, 1.6 mM KCl, 1.24 mM KH_2_PO_4_, 1.3 mM MgSO_4_, 2.0 mM CaCl_2_, 26 mM NaHCO_3_, and 10 mM d-glucose, for 30 min.

### Sampling of labeled neurons from acute slices

**Figure 1:**
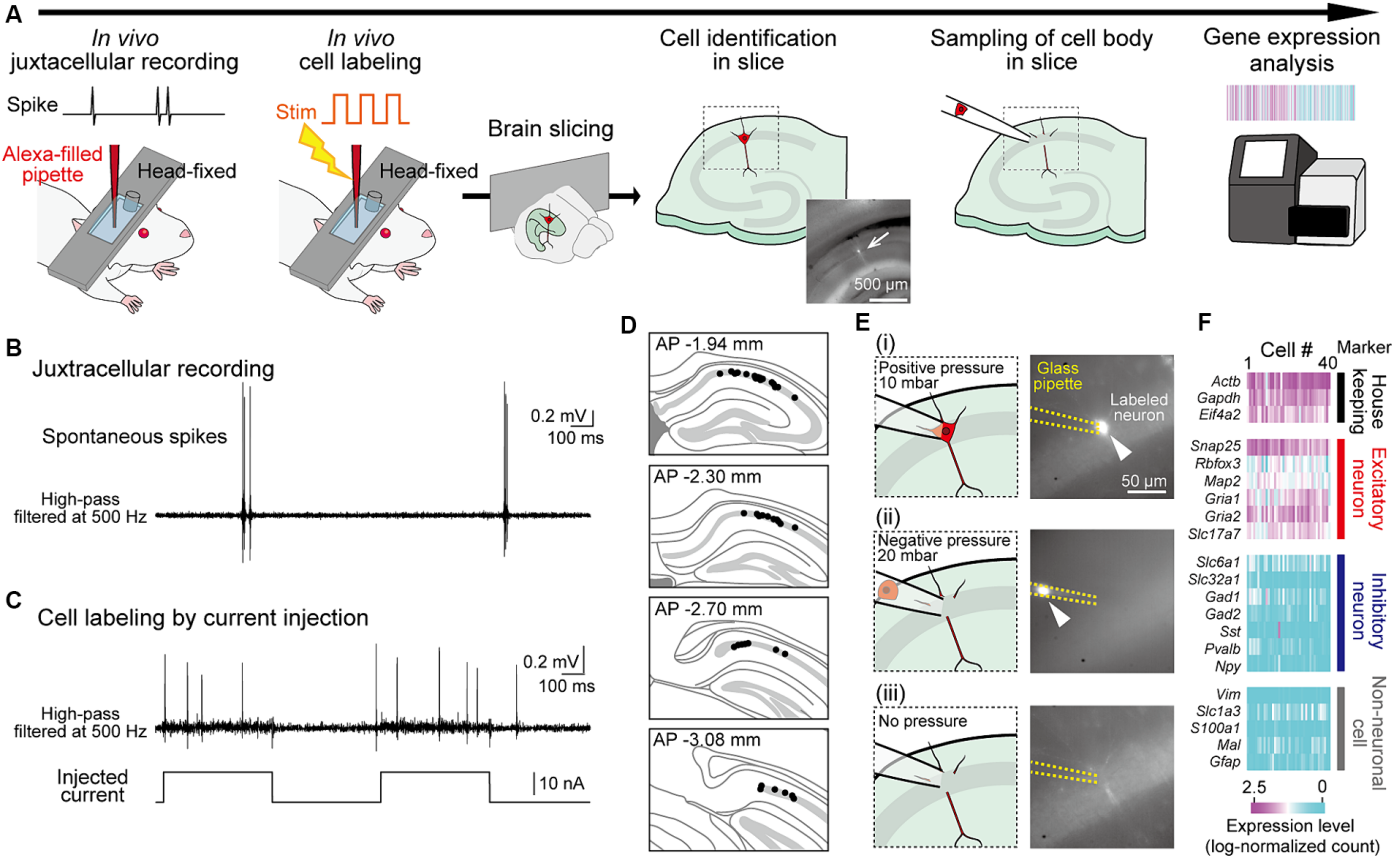
Application of RNA sequencing analysis to juxtacellularly recorded hippocampal neurons from head-fixed mice. **(A)** Experimental procedures. A fluorescently labeled neuron identified in a slice is indicated by an arrow. **(B)** Juxtacellularly recorded high-pass-filtered (500 Hz) voltage trace including spikes. **(C)** Trace showing spikes in response to current injections for labeling of the recorded neuron. **(D)** Superimpositions of the locations of all recorded neurons (black dots) on the dorsal hippocampal CA1 cell layer in sequential coronal brain sections. **(E)** Representative sequential images and fluorescent pictures (from top to bottom) for sampling of a labeled neuron in panel **(C)**. After attaching a glass pipette to the soma of the labeled neuron (i), a negative pressure with 20 mbar is applied to the pipette to suck the cell (ii). After sufficient suction, the pressure was released (iii). **(F)** Log-normalized counts of all 40 neurons sampled, illustrating genes with relevant neuronal markers (housekeeping, excitatory, inhibitory, and non-neuronal cell markers).

The slices were placed in a disposable plastic dish with oxygenated ACSF. Under epifluorescence microscopy (Eclipse FN1, Nikon Solutions Co., Ltd., Tokyo, Japan), the labeled neurons were located from the slices based on the fluorescence of Alexa 488 and/or Alexa 594. After confirming that single neurons, not multiple neurons, were fluorescently labeled, a glass pipette (15–25 μm tip diameter) under very weak positive pressure (<10 mbar) was placed in close contact with the fluorescently labeled neuron (Hempel et al., 2007) (Figure 1E). The intra-solution of the glass pipette consisted of ACSF with 1 U/μL SUPERase•In RNase Inhibitor (Thermo Fisher Scientific, Waltham, MA, USA). Negative pressure (20 mbar) was then applied to the glass pipette, and the soma of the labeled neuron was collected. The glass pipette was carefully withdrawn by maintaining a weak negative pressure to avoid soma loss. Each collected sample was immediately transferred to a lysis buffer (0.111 μM barcoded RT primers, 0.12 mM dNTP mix, 0.3% NP-40, 1 unit/μL RNasin Plus). The samples were stored at −80°C until use.

### Quartz-Seq2 single-cell RNA-seq analysis

The cryopreserved single-cell lysate was used to construct a sequence library in accordance with the methods described in the original Quartz-Seq2 study (Sasagawa et al., 2018). The library was sequenced using an Illumina HiSeq X sequencer (Illumina, San Diego, CA, USA). The sequence specifications of the Quartz-Seq2 library were as follows: Read1, 23 bp (15-bp cell barcode +8-bp UMI); index1, 6 bp; Read2, 91 bp. The Cell Ranger Software Suite v7.1.0 (10x Genomics, Pleasanton, CA, USA) was used to perform sample de-multiplexing, barcode processing, single-cell 3′ unique molecular identifier (UMI) counting and generating the gene-barcode expression matrix. The matrix was imported into Seurat v4 (Hao et al., 2021) for quality control and downstream analyses. Except in certain situations, default parameters were used in all operations. Sctransform in Seurat was used to normalize the UMI count in each sample. Z-scores for gene expression levels were calculated for each gene across all samples.

### Spike analysis

The LFP signals were high-pass filtered at 500 Hz. The envelope of the filtered LFP traces was calculated by Hilbert transformation, and spikes were detected if the peaks of the envelope exceeded a manually defined threshold (0.25–1 mV) so that the spike signals could be separated from the noise. To compute the rise time from the spikes in each neuron, nonfiltered spike signals were aligned to the peak times of the individual spikes, and all aligned filtered traces were then averaged. In the averaged trace, a spike onset was defined as a time when a spike trace first exceeded one standard deviation above the mean of the baseline voltages 0.5–2.0 ms before the peak of the trace. The rise time was calculated as the difference between the onset and peak times.

### Identification of bursty and non-bursty cells

**Figure 2:**
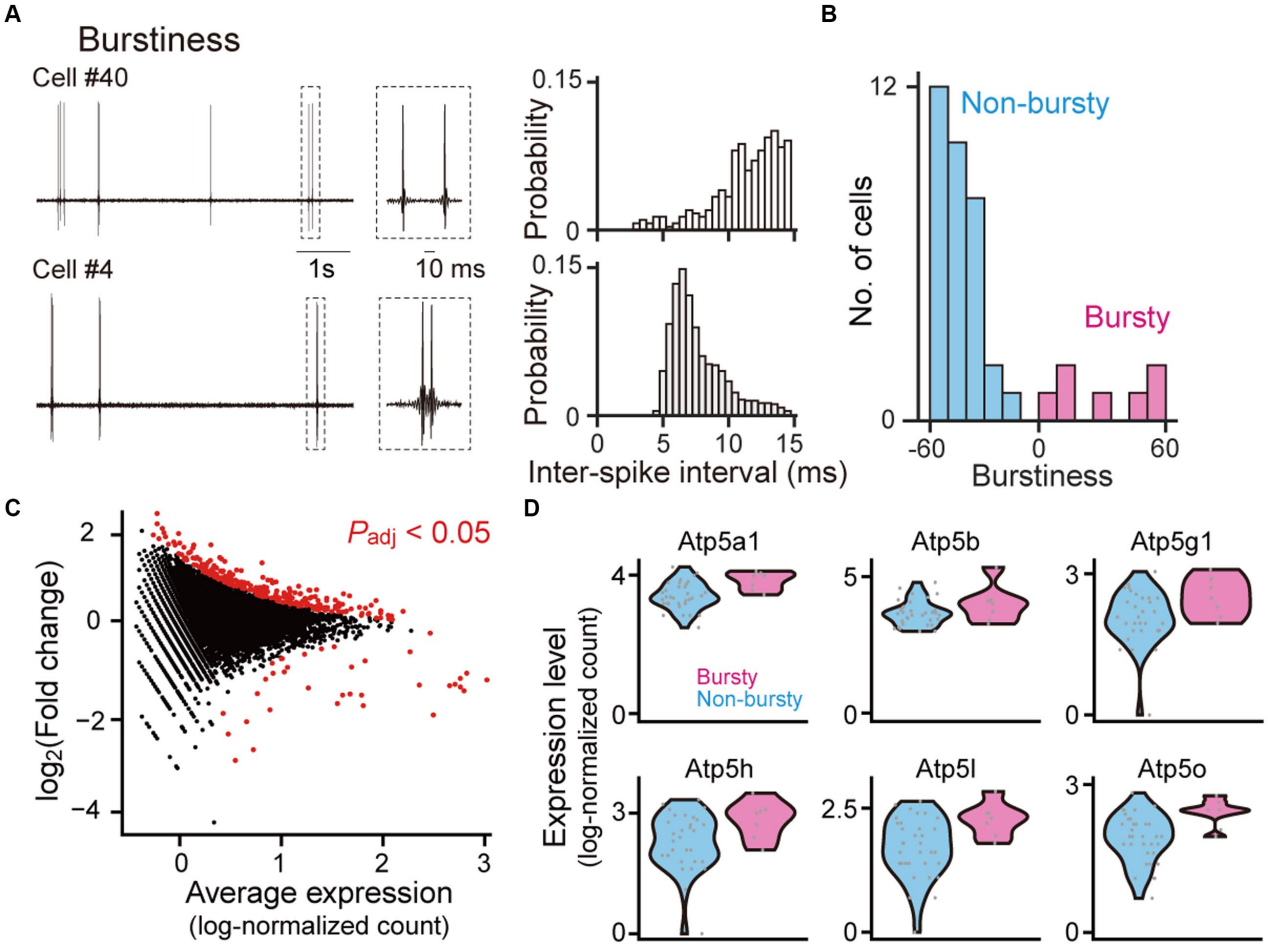
Gene expression profiles between bursty and non-bursty cells. **(A)** (Left) High-pass-filtered (500 Hz) voltage traces of two representative neurons (cells #40 and #4). The rectangle dotted areas are expanded in the right panels. (Right) Histograms of inter-spike intervals computed from the neurons. **(B)** The distribution of burstiness indices from all recorded neurons (*n* = 40 neurons classified into 7 bursty and 33 non-bursty neurons). **(C)** A scatter plot of log2 fold changes against the average expression levels for individual genes. Genes with significant differential expressions between bursty and non-bursty neurons (*P*_adj_ < 0.05, *n* = 292 out of 8,462 genes) are marked in red. **(D)** Violin plots showing the expression levels of Atp5 family genes identified as the differentially expressed genes. Each gray dot represents a single cell. *Atp5a1*: *p* = 1.7 × 10^−10^, *q* = 5.1 × 10^−8^; *Atp5b*: *p* = 1.2 × 10^−18^, *q* = 1.0 × 10^−10^; *Atp5g1*: *p* = 6.0 × 10^−4^, *q* = 0.024; *Atp5h*: *p* = 2.4 × 10^−6^, *q* = 3.1 × 10^−4^; *Atp5l*: *p* = 3.9 × 10^−4^, *q* = 0.0017; *Atp5o*: *p* = 8.0 × 10^−5^, *q* = 6.0 × 10^−3^.

Neurons were classified into bursty and non-bursty neurons, as described previously (Latuske et al., 2015; Ebbesen et al., 2016; Coletta et al., 2018). A principal components analysis was applied to a distribution (i.e., 30-dimensional vector) of inter-spike intervals less than 15 ms (bin = 0.5 ms) (Figure 2A). Neurons were grouped into two clusters, including bursty and non-bursty neurons, through k-means clustering applied to the first three principal components (Supplementary Figure S2). Subsequently, a Fisher’s linear discriminant separating between bursty and non-bursty neurons was computed. For each neuron, a burstiness was defined as a distance from the linear discriminant (Ebbesen et al., 2016). Bursty and non-bursty neurons had positive and negative distance, respectively.

### Hierarchical classification

Based on Word’s minimum variance method and Spearman’s rank correlation coefficient as a measure of similarity in the R software (The R Foundation, Vienna, Austria), hierarchical cluster analysis was performed to group all 8,462 genes and 40 cells according to the degree of similarity present in the gene expression data. In the resulting dendrogram of the genes, we defined 25 metagenes using the tree-cut method implemented through the cuttree function in R software.

### Statistical analysis

R v4.3.0 (R Core Team) and MATLAB 2020a (MathWorks, Natick, MA, USA) were used for statistical analyses. Differentially expressed genes were identified using the ‘poisson’ likelihood ratio test from FindMarkers function in Seurat package. Spearman’s rank correlation coefficients were computed between gene expression levels and rise times or firing rates of individual neurons. The null hypothesis was rejected at *p* < 0.05. To control the false discovery rate in the multiple comparison tests, we calculated the *q*-value using the Benjamini-Hochberg method. The details of the statistical tests are provided in the corresponding legends of the figure panels.

## Results

### Gene expression analysis from juxtacellularly recorded neurons *in vivo*

We designed an experimental approach to measure the gene expression profiles in neurons whose spike patterns were recorded *in vivo* (Figure 1A). To measure neuronal activity in live mice, we applied a juxtacellular recording technique that measures spike patterns at the single-cell level and subsequently labeled the recorded neurons with a fluorophore through electroporation (Pinault, 1994, 1996; Oyama et al., 2013; Dempsey et al., 2015). Mice were anesthetized and their heads were fixed in a stereotaxic device, and a juxtacellular recording was obtained from a dorsal hippocampal CA1 pyramidal neuron using a glass pipette filled with Alexa fluorescent dye (Figure 1B). In a single mouse, we recorded up to four neurons by using two fluorescent dyes and by targeting both sides of the hemispheres. After recording the extracellular voltage signals, including spikes for 28.8 ± 7.2 min (*n* = 40 neurons), electroporation was performed by injecting electrical pulses to introduce the fluorescent dye into the recorded neuron. The injected pulses evoked burst-like spikes with increased baseline voltage fluctuations in the recorded neurons, which are typical signs of successful cell labeling (Figure 1C and Supplementary Figure S1). Immediately after cell labeling, the brains were removed from the mice, and acute hippocampal slices with a thickness of 200 μm were prepared. In the sequence of slices, hippocampal neurons in the CA1 cell layer labeled with fluorescent dye were identified using fluorescence microscopy (Figure 1D and Supplementary Figure S1). The success rate of identifying the labeled neuron was 39.5% (45 neurons / 114 recordings). The labeled neurons were collected using a glass pipette at a negative pressure of 20 mbar (Figure 1E and Supplementary Figure S1). This step was not successful in a minority of neurons (3 / 45 neurons) due to photobleaching. The collected neurons were subjected to single-cell RNA sequencing analysis using the Quartz-Seq2 method (Sasagawa et al., 2018). Two neurons with a read count of less than 5,000 were excluded, resulting in a dataset of transcriptomes from 40 neuron samples (with 50,457 ± 13,599 read counts). In this dataset, all samples were confirmed as excitatory neurons showing stronger expression of housekeeping genes and excitatory neuronal marker genes, but not inhibitory neuronal and non-neuronal marker genes (Figure 1F). Overall, the success rate of total steps from cell labeling to gene expression analysis was 34.5% (40 neurons / 114 recordings).

### Gene expressions between bursty and non-bursty hippocampal neurons

CA1 pyramidal neurons are diverse in the levels of burst firing patterns (Jensen et al., 1996; Jarsky et al., 2008). In our datasets, 40 neurons were classified into 7 (17.5%) bursty and 33 (82.5%) non-bursty neurons based on a PCA and k-means clustering (Latuske et al., 2015; Ebbesen et al., 2016; Coletta et al., 2018) (Figures 2A,B and Supplementary Figure S2), consistent with previous studies (Jensen et al., 1996; Jarsky et al., 2008). Gene expression patterns related to the differences between these two cell types were analyzed (Figure 2C and Supplementary Data S1, *n* = 8,462 genes and 40 cells; adjusted *p*-value <0.05). Overall, 256 and 36 genes showed significantly higher and lower expressions in bursty neurons, respectively. Especially, several genes in the Atp5 family were significantly upregulated in the bursty neurons (Figure 2D; *Atp5a1*: *p* = 1.7 × 10^−10^, *q* = 5.1 × 10^−8^; *Atp5b*: *p* = 1.2 × 10^−18^, *q* = 1.0 × 10^−10^; *Atp5g1*: *p* = 6.0 × 10^−4^, *q* = 0.024; *Atp5h*: *p* = 2.4 × 10^−6^, *q* = 3.1 × 10^−4^; *Atp5l*: *p* = 3.9 × 10^−4^, *q* = 0.0017; *Atp5o*: *p* = 8.0 × 10^−5^, *q* = 6.0 × 10^−3^).

### Gene expressions correlated with firing rates in hippocampal neurons

**Figure 3:**
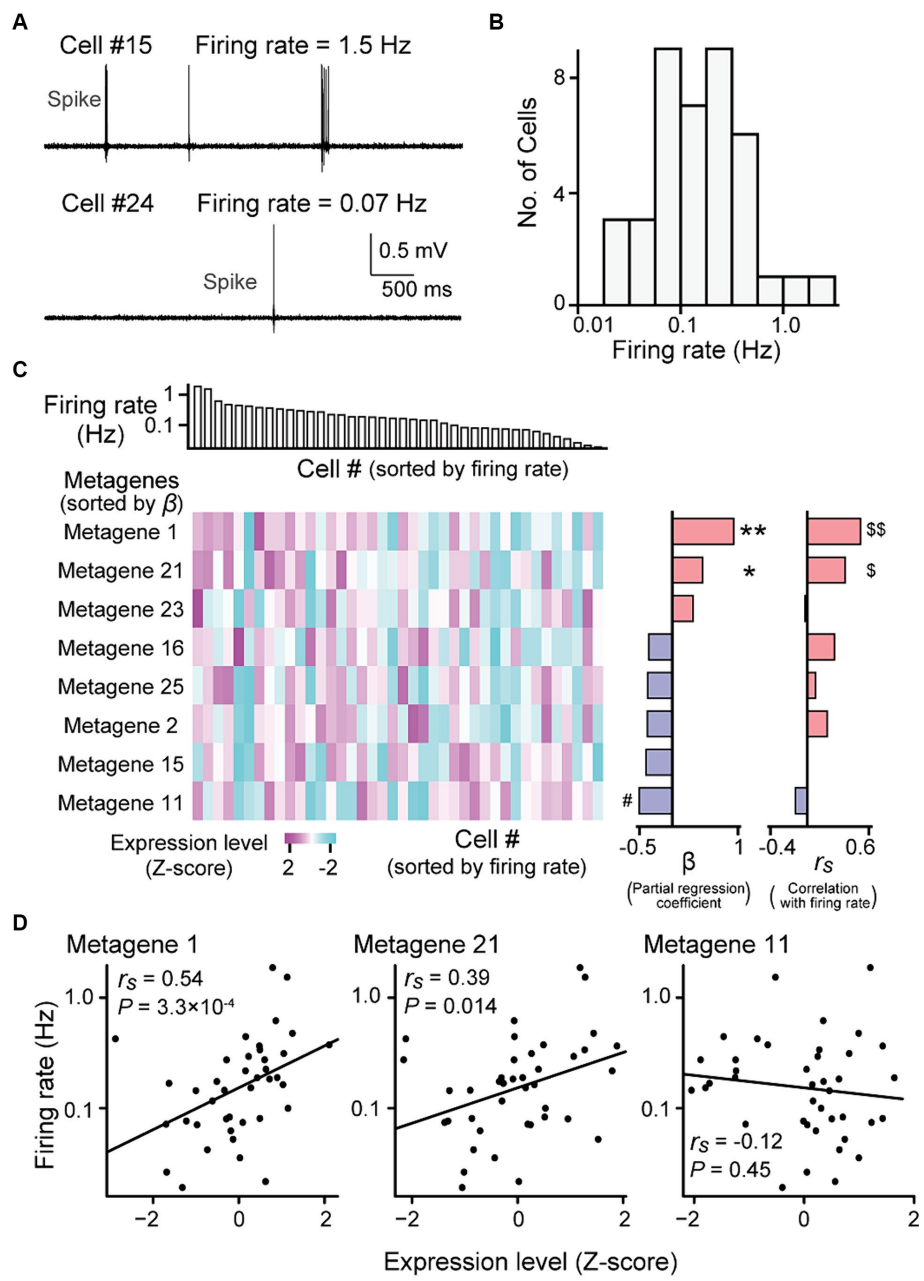
Gene expression profiles correlated with firing rates. **(A)** High-pass-filtered (500 Hz) voltage traces of two representative neurons (cells #15 and #24). **(B)** The distribution of firing rates from all recorded neurons (*n* = 40 neurons). **(C)** (Left top) Neurons were aligned according to their firing rates (*n* = 40 neurons). (Left bottom) A heatmap showing expression levels of metagenes from the individual neurons. Here, the metagenes that used in the multi regression model are shown (see Supplementary Figure S3 and S4 for all 25 metagenes). The metagenes are sorted by their partial regression coefficients (β) computed from the liner regression model (middle). **p = 0.0010, *p = 0.013, ^#^*p* = 0.0033. (Right) Spearman’s rank correlation coefficients (*r_s_*) between their gene expression levels and the firing rates of the individual neurons. ^$$^*q* = 8.4 × 10^−3^, ^$^*q* = 0.18. **(D)** Relationship between the expression levels of metagene 1 (left), 21 (middle) or 11 (right) and the firing rates. Each dot represents a cell (*n* = 40 cells; metagene 1: *r_s_* = 0.54, *p* = 3.3 × 10^−4^, *q* = 8.4 × 10^−3^; metagene 21: *r_s_* = 0.39, *p* = 0.014, *q* = 0.18; metagene 11: *r_s_* = −0.12, *p* = 0.45, *q* = 0.95).

Consistent with previous reports (Mizuseki and Buzsaki, 2013), the firing rates of the hippocampal pyramidal neurons that were recorded using our recording methods varied substantially across neurons, ranging from 0.019 to 1.87 Hz (Figures 3A,B; *n* = 40 neurons). We sought to identify the gene expression profiles associated with these firing rates. All 8,462 genes were classified into 25 metagenic groups based on hierarchical clustering (Supplementary Figure S3 and Supplementary Data S2). The expression level of a metagene was computed as the average of the expression levels of all genes included in the metagene. We applied a multiple regression model fitting in which the contribution of each gene was fitted to the firing rate. This regression analysis showed that metagene 1, 2, 11, 15, 16, 21, 23, and 25 had the maximum contribution rate estimated with the Akaike Information Criterion (*R^2^_adj_* = 0.34, *f* = 3.53, *p* = 0.0054; Figure 3C). In addition, for each metagene, the Spearman’s correlation coefficient (*r_s_*) was computed between the expression levels and firing rates of individual neurons (Supplementary Figure S3). In particular, the expression levels of metagenes 1 and 21 were highly positively correlated with the firing rate (Figures 3C,D right; metagene 1: *r_s_* = 0.54, *p* = 3.3 × 10^−4^, *q* = 8.4 × 10^−3^; metagene 21: *r_s_* = 0.39, *p* = 0.014, *q* = 0.18).

### Gene expressions correlated with rise times of spikes in hippocampal neurons

**Figure 4:**
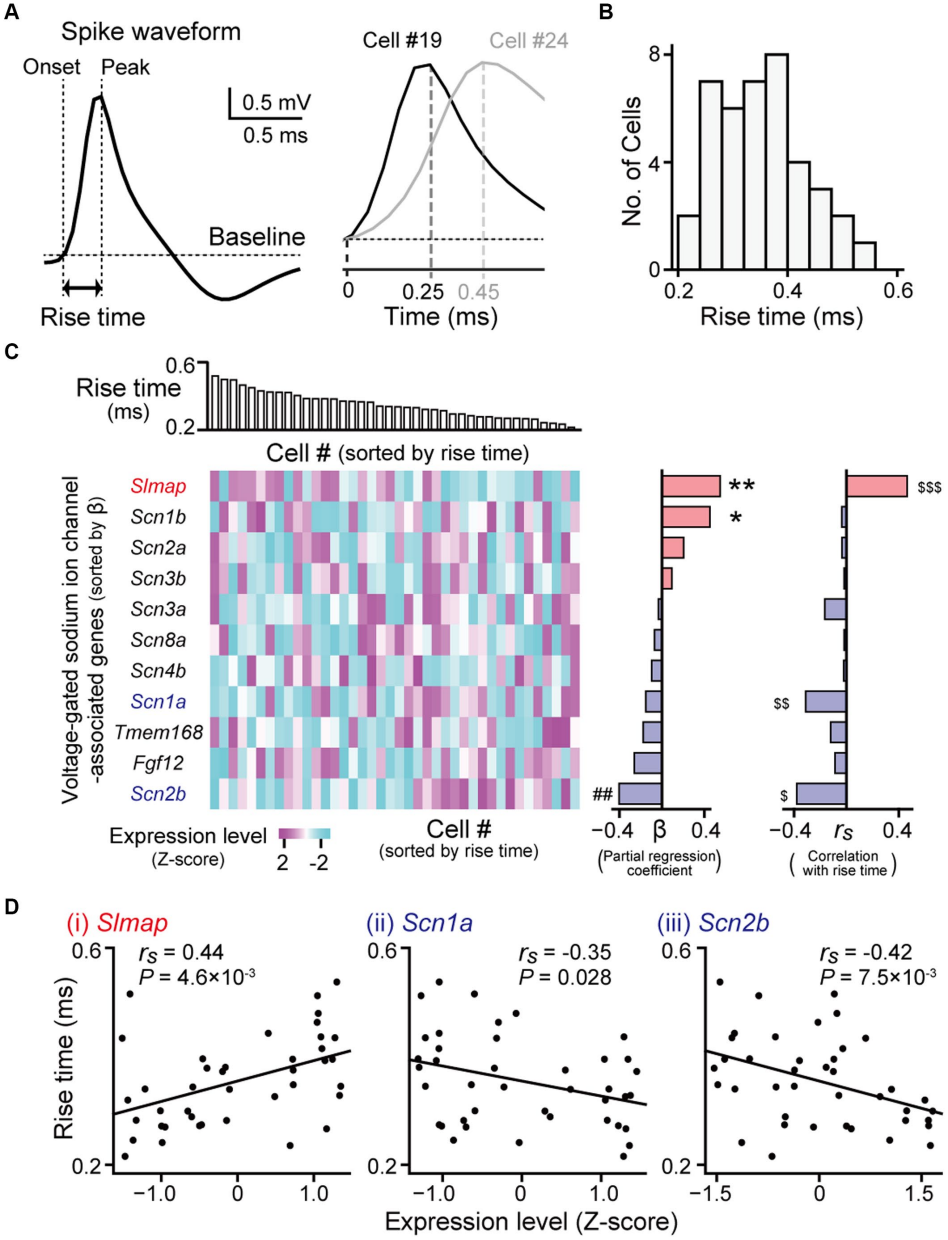
Gene expression profiles correlated with spike rise times. **(A)** (Left) A juxtacellularly recorded spike waveform (high-pass filtered at 500 Hz). (Right) Magnified averaged spike waveforms from two representative neurons (cell #19 and #24). **(B)** The distribution of spike rise times from all recorded neurons (*n* = 40 neurons). **(C)** (Left top) Neurons were aligned according to their rise times. (Left bottom) A heatmap showing expression levels of genes related to voltage-gated sodium ion channels. The genes are sorted by partial regression coefficients (b) computed from the multi regression model (middle). **p = 9.8 × 10^−3^, *p = 0.099, ^##^*p* = 0.080. (Right) Spearman’s rank correlation coefficients (*r_s_*) between their expression levels and the rise times of the individual neurons. ^$$$^*q* = 0.041, ^$$^*q* = 0.10, ^$^*q* = 0.041. **(D)** Three genes showing significant positive or negative correlations (*Slmap*: *r_s_* = 0.44, *p* = 4.6 × 10^−3^, *q* = 0.041; *Scn1a*: *r_s_* = −0.35, *p* = 0.028, *q* = 0.10; *Scn2b*: *r_s_* = −0.42, *p* = 7.5 × 10^−3^, *q* = 0.041). In each graph, the rise times of the individual neurons are plotted against their gene expression levels (*n* = 40 neurons).

Next, we sought to identify genes related to the shape of the spike waveforms. The rise times of the juxtacellularly recorded spikes were computed as the duration between the spike onset and its peak in an averaged spike trace (Figure 4A). Rise times were also substantially variable across hippocampal pyramidal neurons, ranging from 0.22 to 0.54 ms (Figures 4A,B; *n* = 40 neurons). As the rising phases of membrane potentials in spikes are mainly determined by voltage-gated sodium channels with different properties (Westenbroek et al., 1989; Boiko et al., 2001; Mechaly et al., 2005; Katz et al., 2018), we restricted the analysis to genes related to these channels. We obtained the 11 genes from MGI database on AmiGO2 (on 05.09.2023) (Carbon et al., 2009), using “regulation of voltage-gated sodium channel activity” (GO: 1905150) and “voltage-gated sodium channel complex” (GO: 0001518) as queried GO terms. Similar to the analysis of firing rates, we applied a multiple regression model fitting in which the contribution of each gene was fitted to the rise time (Figure 4C, left and middle). This regression analysis confirmed that *Slmap*, *Scn1b*, and *Scn2b* had the maximum contribution rate estimated with the Akaike Information Criterion (*R^2^_adj_* = 0.31, *f* = 6.86, *p* = 9.0 × 10^−4^). In addition, we computed Spearman’s correlation coefficient (*r_s_*) for each gene between its expression levels and the rise times of individual neurons (Figure 4C, right). Of the 11 voltage-gated sodium channel-related genes tested, *Slmap* showed a significant positive correlation between its expression levels and rise times (Figures 4C,D right; *r_s_* = 0.44, *p* = 4.6 × 10^−3^, *q* = 0.041), whereas *Scn1a* and *Scn2b* showed negative correlations (*Scn1a*: *r_s_* = −0.35, *p* = 0.028, *q* = 0.10; *Scn2b*: *r_s_* = −0.42, *p* = 7.5 × 10^−3^, *q* = 0.041). Taken together, these results demonstrate that the expression levels of some genes related to voltage-gated sodium channels are crucially associated with spike rise times in hippocampal pyramidal neurons.

## Discussion

Here, we introduce a series of experiments to identify gene expression profiles from single hippocampal neurons that were recorded using juxtacellular recording in living mice under head-fixed conditions. After recording and labeling the neurons with fluorophores, the labeled neurons were collected on slice preparations. Using these cell samples, we demonstrated that the expression levels of several genes and metagenes were significantly correlated with the burstiness, spike rise times and firing rates of individual neurons.

Hippocampal CA1 pyramidal neurons are classified into two types: bursty neurons and non-bursty neurons (Jensen et al., 1996; Jarsky et al., 2008). Our analysis identified 292 genes that were differentially expressed between these two cell types. In particular, bursty neurons showed significantly higher expressions in genes of the Atp5 family encoding mitochondrial ATP synthase subunits, compared with non-bursty neurons. Previous studies indicated that neurons exhibiting stronger burst firing require more energy than those with weaker firing (Plessy et al., 2008; Moujahid et al., 2014). Consistently, our finding of the higher expression levels of Atp5a family genes in bursty neurons may represent their greater needs for instantaneous energy production.

Among the 25 metagenes tested, metagene 1 showed a significant correlation between its expression levels and neuronal firing rates. For example, metagene 1 was included genes related to microtubule cytoskeleton and regulation of dendrite extension such as *Dscam* (Bruce et al., 2017; Arimura et al., 2020), *Smurf1* (Bryan et al., 2005; Cheng et al., 2011), *Syt17* (Ruhl et al., 2019) (Supplementary Data S2).

We found that two genes related to voltage-gated sodium channel subunits, *Scn2b* and *Slmap*, are associated with the rise times of spikes. *Scn2b* encodes a type I transmembrane protein that regulates the localization of voltage-gated sodium channels (Dulsat et al., 2017; Cortada et al., 2019), leading to alterations of sodium channel currents (Mishra et al., 2011; Chen et al., 2012). *Slmap* encodes a transmembrane protein that shapes action potentials in cardiomyocytes (Ishikawa et al., 2012; Mlynarova et al., 2019). While Slmap has been shown to interact with Scn5a, a sodium channel subtype, this channel subtype is not expressed in the brain. Slmap may thus regulate other types of sodium channels, in the brain. In the Hippo-seq dataset (Cembrowski et al., 2016), these genes are differentially expressed between the superficial and deep layers; CA1 pyramidal neurons in the superficial layer show high expression of Scn2b and low expression of Slmap and those in the deep layer show the opposite tendency. Together with our results, CA1 pyramidal neurons in the deep layer may depolarize more slowly than those in the superficial layer. In the DropViz dataset (Saunders et al., 2018), there are various expression patterns of Scn2b and Slmap genes in the eight clusters of CA1 pyramidal neurons. Notably, clusters #5–3 and #5–4 show contrasting expression patterns in these two genes.

Recently, several methods that utilize optical multicell imaging have been developed to examine how neuronal activity patterns in living mice are linked to gene expression profiles. For instance, *in vivo* two-photon calcium imaging was used to monitor the spike patterns of a neuronal population in the living brain, followed by multiplexed fluorescent *in situ* hybridization to identify several gene expression profiles of the identical neurons (Xu et al., 2020; Bugeon et al., 2022). Another approach involves two-photon calcium imaging, followed by cell sampling of the imaged neurons using pipettes for subsequent gene expression analysis (Liu et al., 2020). Compared with these imaging-based approaches, we used juxtacellular recording techniques to record neuronal spikes, which have unique advantages and disadvantages. First, electrophysiological recordings offer higher temporal resolution (generally tens of kilohertz) than optical imaging, which enables more precise isolation of single spikes within burst firing and a detailed analysis of how they are temporally entrained by extracellular local field potential oscillations, such as hippocampal theta waves and sharp wave ripples. In addition, compared with conventional spatial transcriptomics targeting hundreds of genes in hippocampal neurons (Rodriques et al., 2019; Stickels et al., 2021), substantive cell sampling in our study allowed transcriptome sequences (e.g., Quartz-Seq2) targeting tens of thousands of genes through next-generation sequencers. However, one limitation is the throughput of data sampling because each juxtacellular recording and cell sampling can target only one to two neurons. In addition, there is a possibility that we missed some genes with very few copies from small amount of cell samples. To enhance the throughput of our method, incorporating additional fluorescent dyes with distinct colors, such as Alexa 350 hydrazide, will allow us to increase our attempts of juxtacellular recordings to more than 10 trials per mouse. Furthermore, employing unique molecular identifiers (e.g., barcode labeling) may be effective to circumvent the constraints of cell sampling using fluorescent dyes.

Overall, our method specializes in assessing spike activity patterns on a strict sub-millisecond timescale and further uncovers their comprehensive gene expression profiles in juxtacellularly recorded neurons. Because recording electrodes can access nearly all brain regions, including deep brain areas that are difficult to access by optical imaging, our method is applicable not only to hippocampal pyramidal cells but also to a variety of brain regions and neuron types. In addition, by taking advantage of the applicability of juxtacellular recordings in freely moving animals (Herfst et al., 2012; Tang et al., 2014), our method is expected to be valuable for unveiling how behavior-relevant spike patterns are associated with the gene expression profiles of individual neurons.

## Data availability statement

The gene expression dataset presented in the study is deposited in the Gene Expression Omnibus, accession number GSE262930.

## Ethics statement

The animal study was approved by the Experimental Animal Ethics Committee at the University of Tokyo (approval number: P29-7) The Committee on Animal Experiments at Tohoku University (approval number: 2022 PhA-004). The study was conducted in accordance with the local legislation and institutional requirements.

## Author contributions

HY: Conceptualization, Data curation, Formal analysis, Funding acquisition, Investigation, Methodology, Resources, Visualization, Writing – original draft, Writing – review & editing. YG: Data curation, Funding acquisition, Resources, Writing – original draft, Writing – review & editing. KO: Methodology, Writing – review & editing. NA: Formal analysis, Writing – review & editing. YI: Supervision, Writing – review & editing. TS: Conceptualization, Funding acquisition, Project administration, Supervision, Visualization, Visualization, Writing – review & editing.
