# Supplementary material for: A method to analyze gene expression profiles from hippocampal neurons electrophysiologically recorded in vivo
Source: Front Neurosci. 2024 Apr 17;18:1360432. doi: 10.3389/fnins.2024.1360432 (PMC11061373; doi:10.3389/fnins.2024.1360432)
Supplement: Supplementary file 3 [file Data_Sheet_3.DOCX]

Supplementary Material

# Supplementary Data

**Supplementary Data 1.** The full list of differentially expressed genes (*P*_adj_ < 0.05) between bursty and non-bursty neurons. The first column shows the name of the gene, the second column shows the log_2_ fold change, and the third column shows the adjusted *P*-value.

**Supplementary Data 2.** Genes within each metagene. The first column shows the names of the genes, and the second column shows the metagene ID.

#
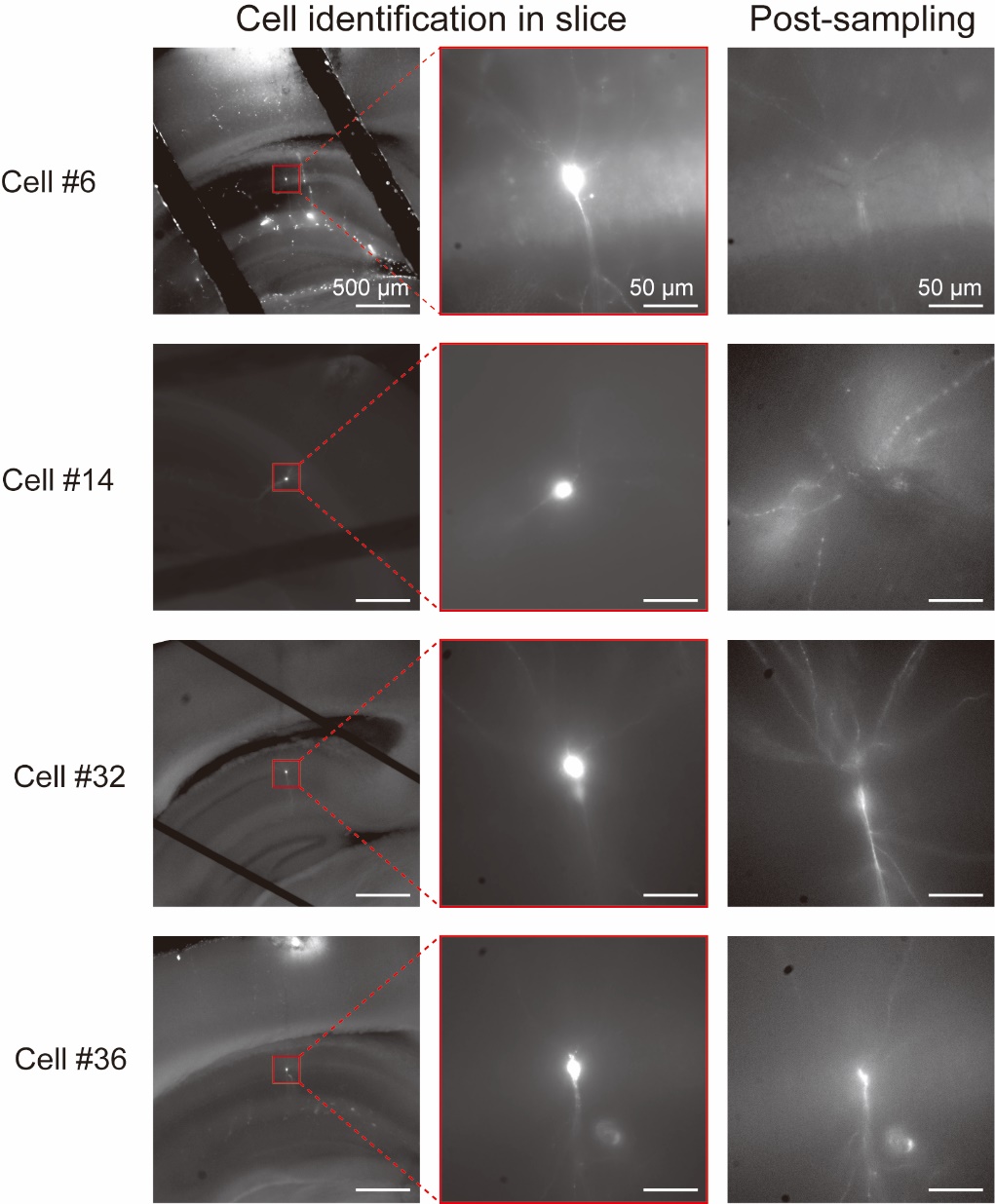
Supplementary Figures

**Supplementary Figure 1.** Additional example of identification and collection of the recorded neurons.

**
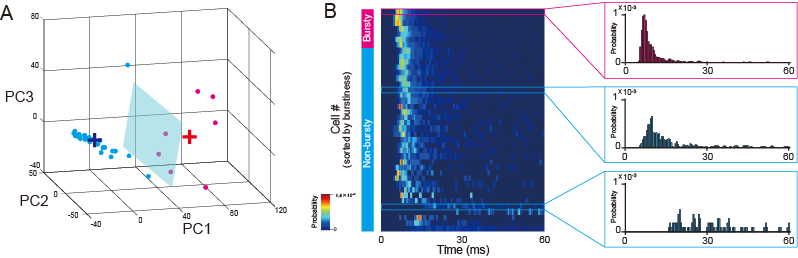
Supplementary Figure 2.** Classification as bursty or non-bursty based on their inter-spike interval histograms. (A) A 3D plot of principal components with “+” representing the center of each cluster. The blue boundary plane classifies non-bursty (cyan) and bursty neurons (magenta). (B) A heatmap showing inter-spike intervals of all recorded neurons, sorted by their burstiness value.

**
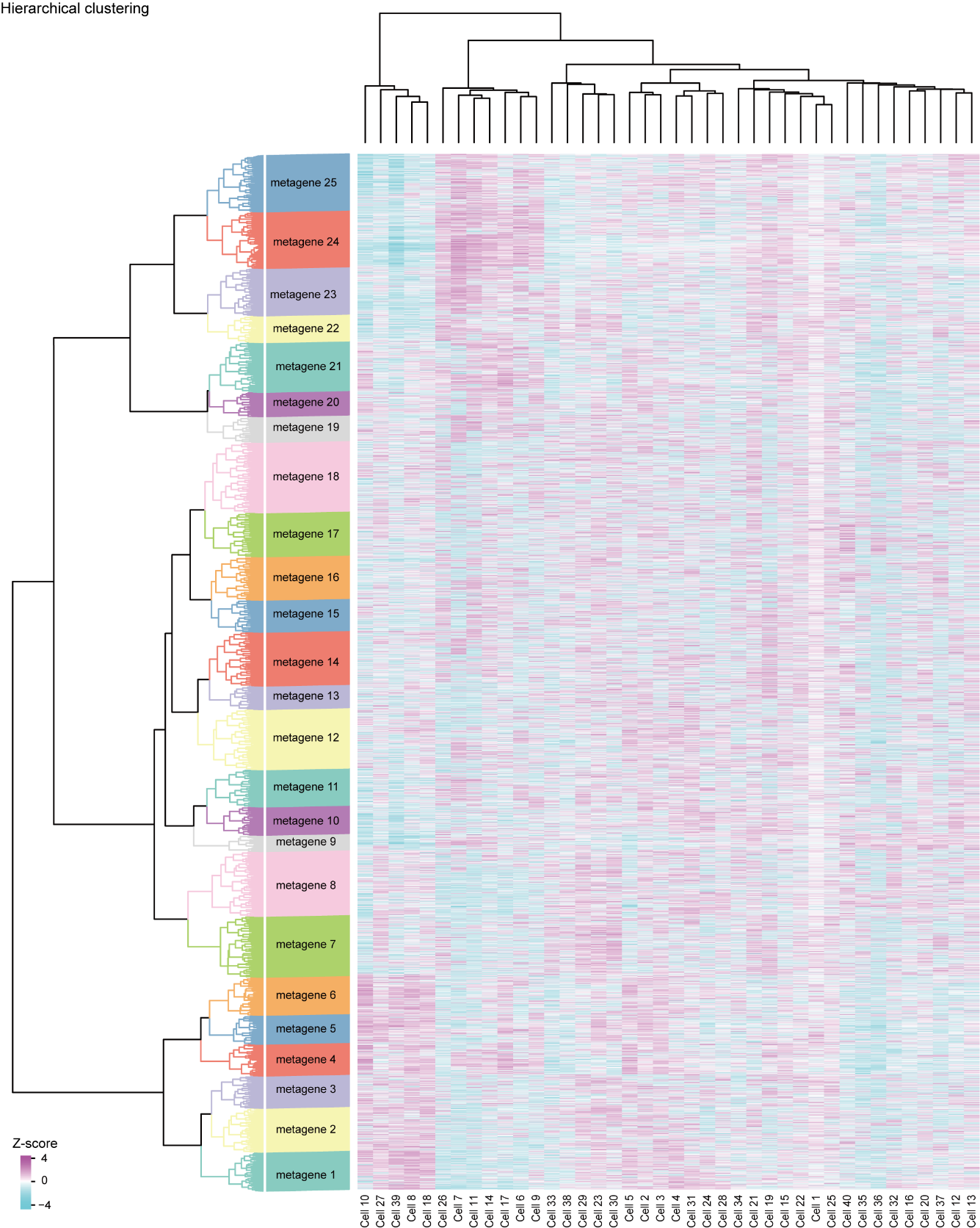
Supplementary Figure 3.** All metagene datasets shown in Figure 3C. A heatmap summarizing the Z-score expression levels of all 25 metagenes and the corresponding dendrogram identified from hierarchical clustering (40 cells and 8462 genes).

**
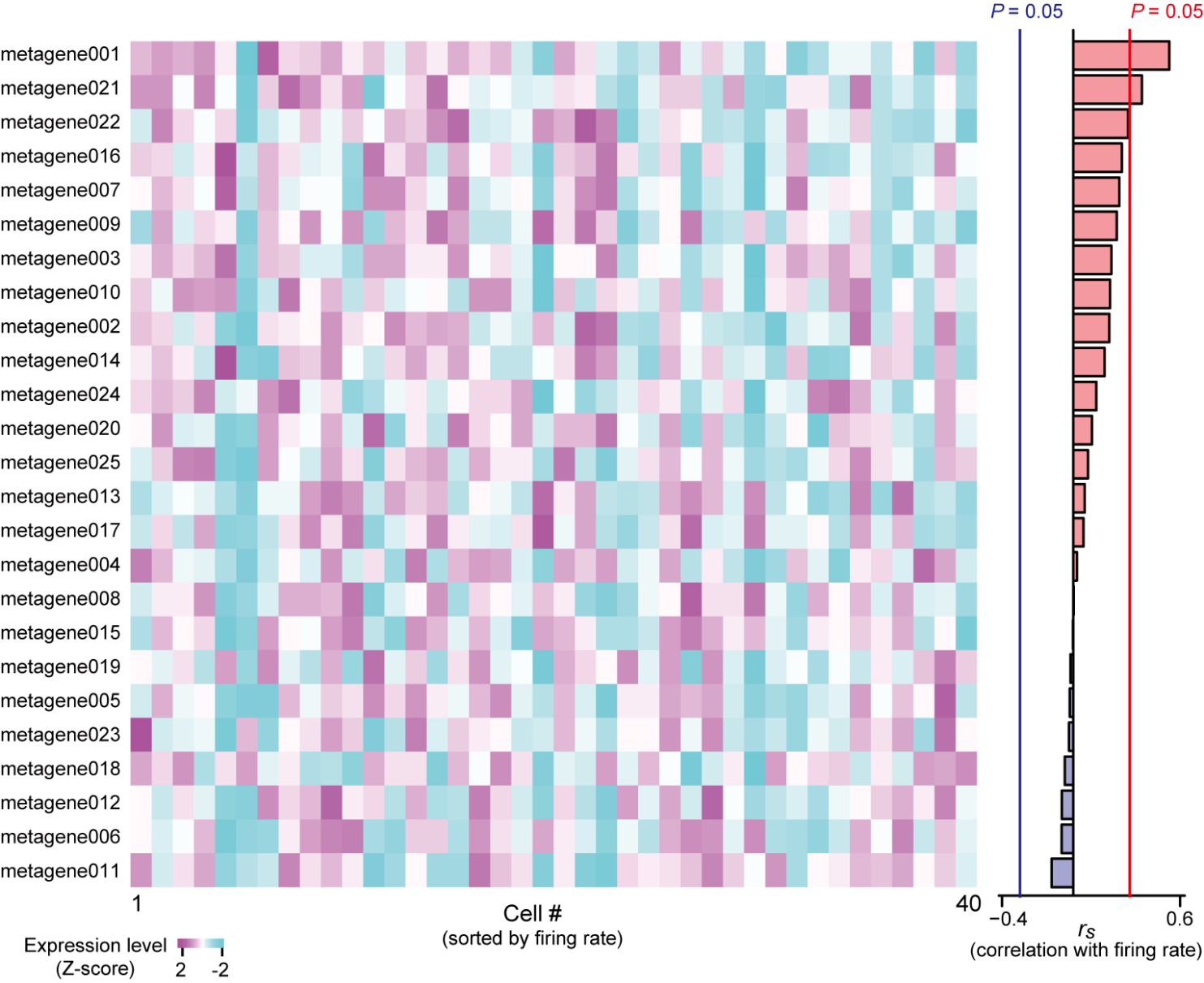
Supplementary Figure 4.** All metagene datasets shown in Figure 3C. A heatmap showing the expression of all 25 metagenes in individual neurons (*n* = 40). The metagenes were sorted using Spearman’s rank correlation coefficients (*r_s_*) between their expression levels and the firing rates of individual neurons (right). The red and blue lines indicate the thresholds with *P* = 0.05 to define significant positive and negative correlations, respectively.
